# Synergistic Flame-Retardant Mechanism of Dicyclohexenyl Aluminum Hypophosphite and Nano-Silica

**DOI:** 10.3390/polym11071211

**Published:** 2019-07-19

**Authors:** Heng Zhang, Junliang Lu, Hongyan Yang, Heng Yang, Jinyan Lang, Qinqin Zhang

**Affiliations:** 1 College of Marine Science and Biological Engineering, Qingdao University of Science & Technology, Qingdao 266042, China; 2 Key Laboratory of Biomass Chemical Engineering of Ministry of Education, Zhejiang University, Hangzhou 310027, China

**Keywords:** nano-silica, ADCP, synergetic flame-retardant, PA66

## Abstract

The flame retardant dicyclohexenyl aluminum hypophosphite (ADCP) and nano-silica are added to PA66 to improve flame retardant property of the composite. The flame-retardant property of the composite is tested via oxygen index test, vertical burning test, and cone calorimetry test. Combustion residues are tested using scanning electron microscopy, EDS spectroscopy, and Fourier infrared analysis. Results show that flame-retardant ADCP can effectively promote the formation of a porous carbon layer on the combustion surface of PA66. Nano-silica easily migrates to the material surface to improve the oxidation resistance of the carbon layer and the density of the carbon layer’s structure. It can also effectively prevent heat, flammable gases, and oxygen from entering the flame zone and enhance the flame retardant properties of ADCP.

## 1. Introduction

Polymers are increasingly being used in manufacturing and everyday life, but their fire risks are causing concerns. When polymers are burning, they are thermally induced to decompose (pyrolysis) into smaller pieces. Then these smaller pieces volatilize and burn with oxygen. This kind of combustion is mainly affected by four aspects: heat source, oxygen, fuel, and free-radical chain reaction. The combustion process can release more heat, which radiates to the unburnt material and continues to promote pyrolysis and combustion until the heat/fuel/oxygen runs out. To effectively inhibit or delay the burning of the substrate and reduce the risk of fire, a flame retardant is generally required in the polymer [1,2,3].

Alkyl hypophosphite is a new flame retardant that has good thermal stability and high efficiency. The materials formed by mixing with polymers have good mechanical property, electrical property, and processability [4]. Alkyl hypophosphite has high phosphorus content and can fully exert the flame-retardant characteristics of phosphorus-based flame retardants. It also has good flame-retardant effects in the condensed and gas phases [5,6]. When the phosphorus of alkyl hypophosphite has the same valence state, the flame-retardant effect is affected by the groups around the phosphorus atom and the ionic bond [7]. Zhao et al. [8] find that the flame-retardant effect on PA6 is optimal when aluminum hypophosphite and aluminum isobutylphosphinate have a weight ratio of 1:1 (15 wt% of the total). The substrate oxygen index obtained is 28.3%. Cao et al. [9] study the synergistic flame-retardant effect of 1,1′-bis(4-hydroxyphenyl)-methylene-bis (9,10-dihydro-9-oxa-10-phosphaphenanthrene-10-oxide-2-hydroxypropan-1-yl) and aluminum diethylphosphinate (AlPi) composites on glass fiber reinforced polyamide 66 (PA66). With a mass ratio of 1:1, the substrate has a high limiting oxygen index (LOI) value and low total heat release (THR) value, which proves its good synergistic flame-retardant effect. He et al. [10] fuse aluminum diisobutylphosphinate, organically modified layered montmorillonite (OMMT), and substrate polyamide 6 (PA6) to obtain a composite material with excellent flame-retardant and mechanical properties. The morphological structure and composition analysis of the combustion residue prove that OMMT promotes the formation of a full, dense, and uniform carbon layer on the material surface and increases the strength of the coke layer during combustion.

When the alkyl phosphinate flame retardant and the nano-material are proportionally compounded into the polymer substrate, a strong synergistic flame-retardant effect arises. Ma et al. [11] prepare a new aluminum-organophosphorus hybrid nano-rod by reacting aluminum hydroxide with dibenzylphosphinic acid, which can greatly improve LOI value with epoxy resin. Si et al. [12] study the synergistic flame-retardant effect of nano-Sb_2_O_3_ and AlPi in polyethylene terephthalate. Results show a signficant synergy between the two, and the LOI value increases to 33.6% when the mass ratio between AlPi and nano-Sb_2_O_3_ ranges from 1:1 to 4:1 (4 wt%, 10 wt% of the total, respectively). Gallo et al. [13] add AlPi and nano-Fe_2_O_3_ to poly (butylene terephthalate) and find the complementary effect of 5 wt% AlPi and 2 wt% metal oxide. The material burn rating is V-0. In this process, a small amount of nano-Fe_2_O_3_ promotes the condensed phase to form carbon, thereby improving the flame-retardant efficiency of AlPi.

In conclusion, flame-retardant ADCP can effectively promote the formation of a porous carbon layer on the combustion surface of PA66, and nano-silica can make the carbon layer structure compact. Carbon layer acts as a flame retardant by isolating heat and air. The flame-retardant mechanism of ADCP is mainly manifested in two aspects: condensed phase flame retardant and gas phase flame retardant. The flame-retardant mechanism of nano-silica is mainly manifested in promoting the formation of the condensed phase. In contrast to the flame-retardant system with nano-silica, the loose and rough carbon layer formed by PA66 without nano-silica can cause the combustible gas to enter the flame zone through the pore structure. Hence, the effect is inferior to the system added nano-silica. A compact carbon layer structure can effectively prevent heat transfer, and reduce inflammable and combustion-supporting gases enter the flame zone. It can also effectively prevent the formation of droplets and the further spread of flames. Therefore, nano-silica can work with ADCP to build an efficient flame-retardant system. The main purpose of this paper is to introduce the new flame retardant and its synergistic flame-retardant mechanism with nano-silica, it will serve as a reference for the future development and application of dicyclohexenyl aluminum hypophosphite flame retardant.

## 2. Materials and Methods

### 2.1. Reagents and Equipment

The reagent used comprised PA66 particles (DuPont Corporation, Midland, MI, USA, industrial grade), nano-silica (Aladdin reagent, nano-level), and dicyclohexenyl aluminum hypophosphite (laboratory made, nano-level).

The equipment used included a mixer (SU-70B, Changzhou Suyan Technology Co., Ltd., Changzhou, China), precision automatic tablet press (2G-10T, Dongguan Zhenggong Mechanical and Electrical Equipment Technology Co., Ltd., Dongguan, China), oxygen index measuring instrument (JF-3, Nanjing Jiangning District Analytical Instrument Factory, Nanjing, China), horizontal and vertical combustion measuring instrument (CZF-3, Nanjing Jiangning District Analytical Instrument Factory, Nanjing, China), scanning electron microscope (JSM-6700F, Japan Electronics Corporation, Tokyo, Japan), infrared spectrometer (510P, Neaspec Corporation, Munich, Germany), cone calorimeter (Standard, Fire Test Technology Co., Ltd., West Sussex, UK), energy spectrometer (INCA, Oxford Co., Ltd., Oxford, UK), and gold spray instrument (JFC-1600, Japan Electronics Corporation, Tokyo, Japan).

### 2.2. Sample Preparation

Dicyclohexenyl aluminum hypophosphite powder was prepared according to the literature [14,15]. The sodium hypophosphite (0.1 mol) and cyclohexene (0.3 mol) were placed in a four-necks flask. With the condensation tube and the nitrogen protection, water bath was mixed for heating reaction. The benzoyl peroxide was dissolved with the amount of solvent acetic acid by dropwise addition of the reaction control, the reaction temperature and reaction time were controlled to obtain the dicyclohexyl phosphinic acid sodium. The product was added with deionized water and vacuum distilled for three times to remove the unreacted excess cyclohexene. Approximately 25 mL of aluminum sulfate solution with a mass concentration of 20% was subsequently added. The temperature was maintained at 40 °C, and the reaction time was 3 h. Afterward, we obtained white suspension, which was washed three times with deionized water, filtrated, dried to obtain the dicyclohexyl phosphinic acid aluminum white solid powder. The product structure was determined by 1H NMR and phosphorus spectra. The product yield was 86%.

Prior to the start of the experiment, PA66 particles, dicyclohexenyl aluminum hypophosphite flame retardant and nano-silica were dried in a vacuum oven at 80 °C for 10 h to remove excess water. The PA66 particles were melted in an internal mixer at 250 °C, then a certain amount of dicyclohexenyl aluminum hypophosphite was added, thoroughly mixed for 10 min, and afterwards a certain amount of nano-silica was added, and the three were blended for 10 min to make the flame retardant. The agent was uniformly dispersed in the matrix. Finally, the sample material from the internal mixer was placed in a mold, placed on a flat vulcanizer, and pressed into a polymer calendered sheet at 260 °C and 10 MPa (with the size of 100 × 100 × 3 mm^3^). The time was usually 10 min.

### 2.3. Flame-Retardant Performance Test

The limiting oxygen index was measured using the JF-3 oxygen index tester, and the polymer tablet was cut into a certain specification spline using a woodworking band saw machine with the following dimensions: 120 mm × 6.5 mm × 3.0 mm [16,17,18]. According to GB/T2406.1-2008, “Plastics Using Oxygen Index Method to Determine the Combustion Behavior” were tested. The vertical combustion test was carried out using the CZF-3 horizontal vertical burning tester, and the 100 mm × 13 mm × 3.2 mm spline was tested according to the national standard “Measurement Method of Plastic Burning Performance and Vertical Method GB/T2408-2008” [19]. Based on the “Test Method for Heat Release Rate of Building Materials GB/T16172-2007/ISO5660-1:2002,” the sample was measured at a heat radiation power of 35 kW/m^2^ using a standard cone calorimeter. The sample size was 100 mm × 100 mm × 3 mm. During LOI and vertical burning test, every datum was measured 5 times and took the average value.

After the limiting oxygen index was determined, the carbon residue sample of the combustion section was fixed on the sample stage with a conductive paste and the surface was sprayed with thin gold coating. The morphology of the sample was analyzed using scanning electron microscopy (SEM) to observe the surface morphology of the gold-plated sample. The burned coke residue was dried, and the infrared spectrum of the sample was measured by a potassium bromide tableting method. The dried sample was taken 1~2 mg and equably mixed with 200 mg of pure potassium bromide crystal particles. After they were grinded uniformly and pressed into a transparent thin sheet, placed in a 510-type FTIR infrared spectrometer for infrared spectroscopy determination. Finally, the burned coke residue was dried, and the part sample was coated with thin gold coating by sputtering. The sample was then placed in an EDS detection spectrometer for detection and elemental determination of the carbon layer.

## 3. Results and Discussion

### 3.1. Formulation and Combustion Properties of PA66 and Its Composites

The experimental results showed that the PA66 pure sample was easy to ignite and had a LOI value 21.5%. The UL-94 test was V-2. When only 15% ADCP was added, the LOI value of PA66 increased to 32%, the combustion level reached V-0, and ADCP produced a flame-retardant effect. When the amount of flame retardant was reduced to 12 wt%, the flame-retardant effect was reduced. The oxygen index of the flame-retardant composite material was also reduced to 30.5%. Thus, the amount of ADCP added in a certain range directly affected the flame-retardant effect. When only 1 wt% of nano-silica was added, the LOI value of the composite increased to 31.7%. The flame-retardant effect was close to add 3 wt% ADCP. Table 1 showed that the LOI value of the PA66 composited increased with the addition of nano-silica, and the increase could be greatly improved when ADCP and nano-silica were added in a proper ratio within a certain range. According to these results, a flame-retardant PA66 material was preferably prepared by 12%ADCP/3%nano-silica. The data details are shown in Table 1.

### 3.2. Cone Calorimeter Analysis

**Figure 1:**
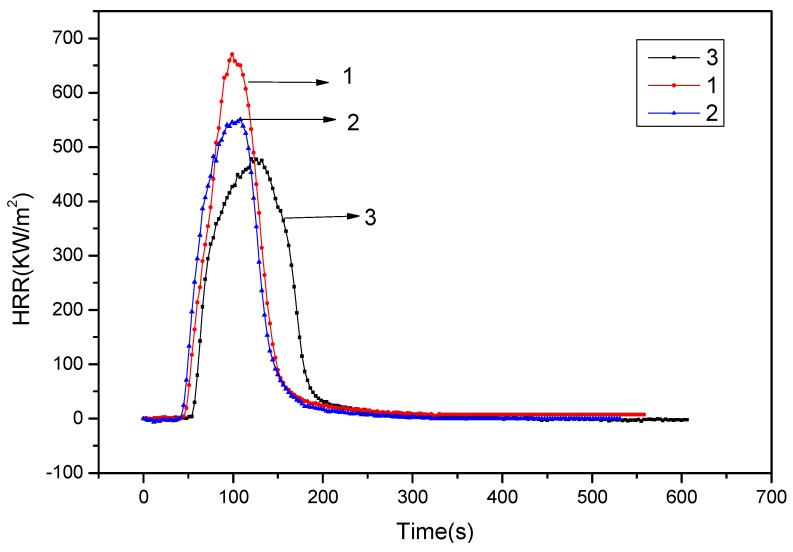
HRR curves of PA66 and the FR-PA66: 1-PA66; 2–15% ADCP FR-PA66; 3–12% ADCP/3% Nano-silica FR-PA66.

**Figure 2:**
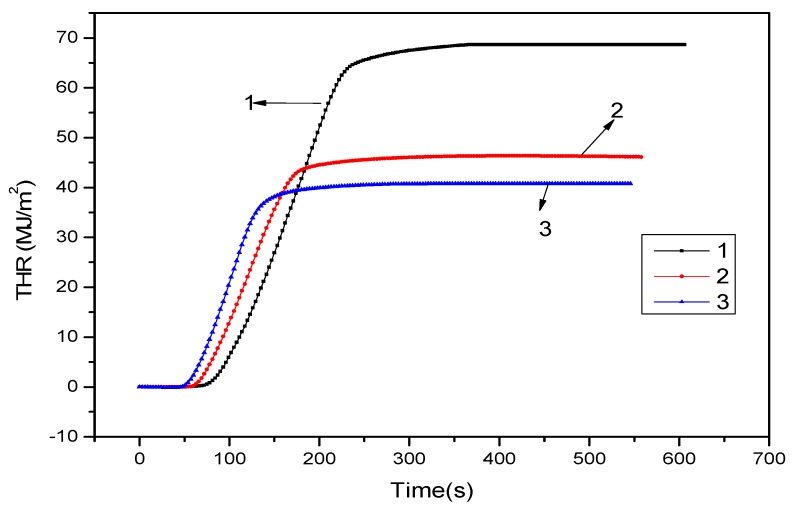
THR curves of PA66 and FR-PA66: 1-PA66; 2–15% ADCP FR-PA66; 3–12% ADCP/3% Nano-silica FR-PA66.

Figure 1 and Figure 2 showed the heat release rate (HRR) and THR data of pure PA66, 15% ADCP FR-PA66, and 12% ADCP/3% nano-silica FR-PA66 at 35 kW/m^2^ thermal radiation power. The HRR of 15% ADCP flame-retardant PA66 decreased, with a peak value and total HRR of 546.5 kW/m^2^ and 46.3 MJ/m^2^, respectively. The peak HRR and THR of 12% ADCP/3% nano-silica FR-PA66 were 462.2 kW/m^2^ and 40.7 MJ/m^2^, respectively. These values further decreased. Therefore, the flame-retardant ADCP could substantially reduce the HRR and THR rate of PA66. The HRR and THR of the PA66 composite material continued to decrease, and the time to reach the peak of the HRR was prolonged after an appropriate amount of nano-silica was added. This effect was caused by the dicyclohexenyl aluminum hypophosphite in the condensed phase. The addition of nano-silica enhanced the charring ability of the flame-retardant system. The carbon layer formed prevented heat, oxygen, and flammable gases from entering the flame zone. In addition, it prevented flame development and further combustion, and reduced heat release, thus achieving a flame-retardant effect.

Table 2 showed the peak of mass loss and smoke generation rates. The rate of mass loss reflected the intensity of material combustion. As shown in Table 2, the peak mass loss rate of the PA66 composite with ADCP flame retardant was substantially lower than that of pure PA66 sample. This finding showed that ADCP promoted char formation. The peak mass loss rate of ADCP flame retardant PA66 with nano-silica added was lower than that of ADCP flame-retardant system without nano-silica. Therefore, nano-silica could further promote the formation of a carbon layer, isolate heat oxygen, and prevent heat transfer. In terms of smoke generation rate, the addition of ADCP flame retardant substantially reduced the peak smoke generation rate of PA66 composites, and the peak value further decreased to 0.09 m^2^/s after adding nano-silica. Thus, an appropriate amount of nano-silica could produce a clear smoke suppression effect due to the promotion of nano-silica to the carbon formation of PA66 and the increased density of the carbon layer.

### 3.3. SEM and EDSanalysis after Combustion

**Figure 3:**
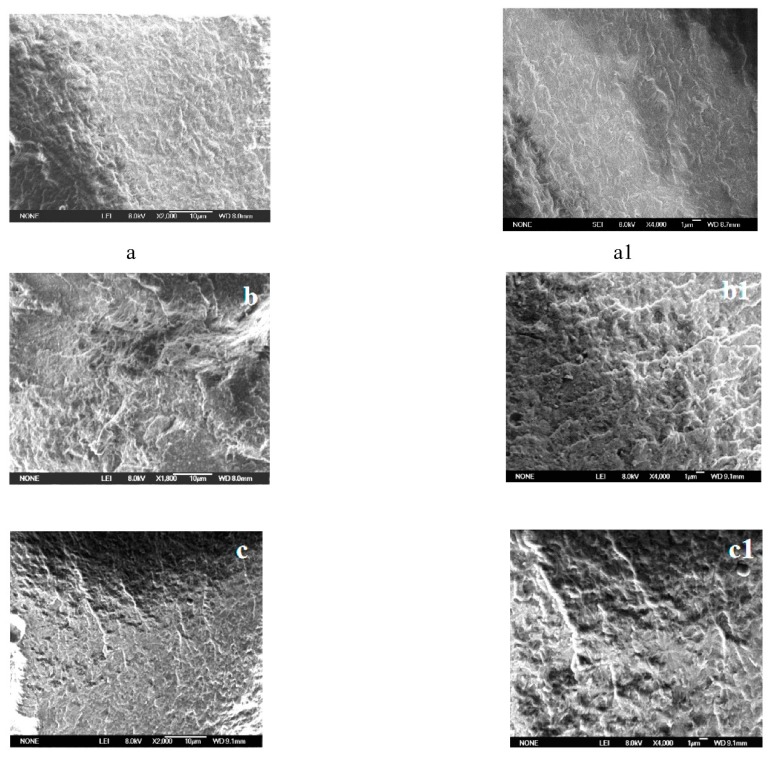
SEM photographs of the carbon layer after cone calorimetry test. (**a**, **a1**-PA66; **b**, **b1**-PA66/15% ADCP; **c**, **c1**-PA66/3% Nano-silica/12% ADCP).

To explain the synergistic flame-retardant effect of nano-silica and dicyclohexenyl aluminum hypophosphite via a microscopic view, the carbon layer after the experiment was analyzed via SEM. The result is shown in Figure 3.

Figure 3 illustrated the microstructure of the carbon layer after cone calorimetry test, in which (a, a1) PA66 was the pure sample, (b, b1) PA66/15% ADCP and (c, c1) PA66/3% nano-silica/12% ADCP. Among them, the picture on the right was a further enlargement in each group. In contrast to (a, a1) the smooth surface, (b, b1) the uneven surface was obviously rough and contains holes, which represented the porous loose carbon layer formed during the combustion process of the flame retardant. In the comparison of (b, b1) and (c, c1), (c, c1) had a smoother surface, because nano-silica could effectively fill the aperture of rough carbon layer. The flame retardant PA66 with nano-silica had a compact carbon layer structure.

**Figure 4:**
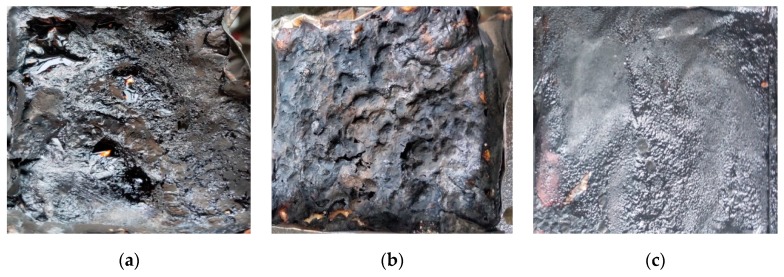
Photographs of sample after the cone test: (**a**) PA66; (**b**) PA66/15% ADCP; (**c**) PA66/12% ADCP/3% Nano-silica.

Figure 4 showed the macrostructure of the sample after cone calorimetry. The flake charcoal residue of PA66 was fractured, and the charcoal layer structure of PA66 with ADCP was fluctuant after burning. The carbon layer structure of PA66 with ADCP and nano-silica was flat and slightly fluctuated.

**Figure 5:**
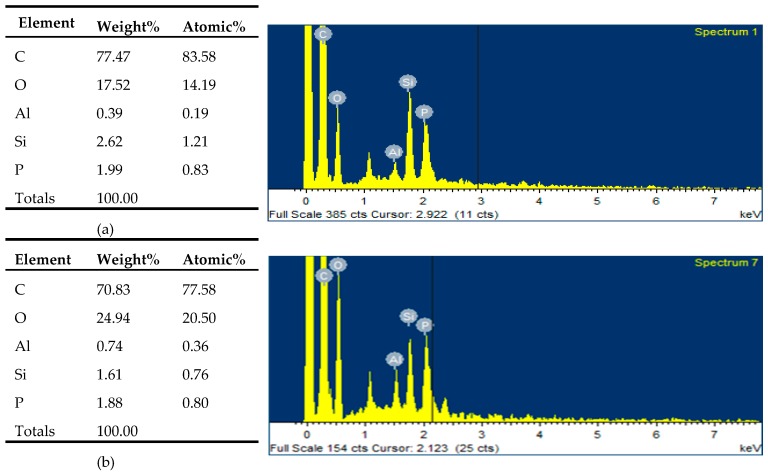
Various element contents and EDS results of products: (**a**) surface of the carbon layer; (**b**) section of the carbon layer.

Figure 5 was an energy dispersive spectroscopy (EDS) of the surface and cross section of the residue after the cone calorimetry experiment. EDS analysis showed that the carbon content on the surface of the carbon layer was higher than that on the cross section because of combustion and carbonization on the surface of the material. The content of silicon element on the surface of carbon layer was higher than that on the cross section because of the flow of macromolecule materials in the high-temperature combustion environment. This proved that nano-silica was easy to migrate to the material surface when the material burned. Based on this phenomenon, we believed that the migration of nano-silica was affected by polymer flow. As the material burned, nano-silica migrated with large molecules due to intermolecular forces. When some of the large molecules moved to the surface of the material, the high temperature caused them to decompose and form carbon layer. The nano-silica located in them and participated in the formation of carbon layer. It made the carbon layer denser and prevented the formation of droplets. In this process, carbon on the surface of the material and nano-silica were both subjected to intermolecular forces and formed chemical bonds, which prevented carbon from reacting with oxygen in the air in a way. Therefore, nano-silica with tiny structure could be easily transferred to the material surface and improved the antioxidant property of carbon layer in the pa66 flame retardant system.

### 3.4. Fourier Infrared Analysis and Detection

**Figure 6:**
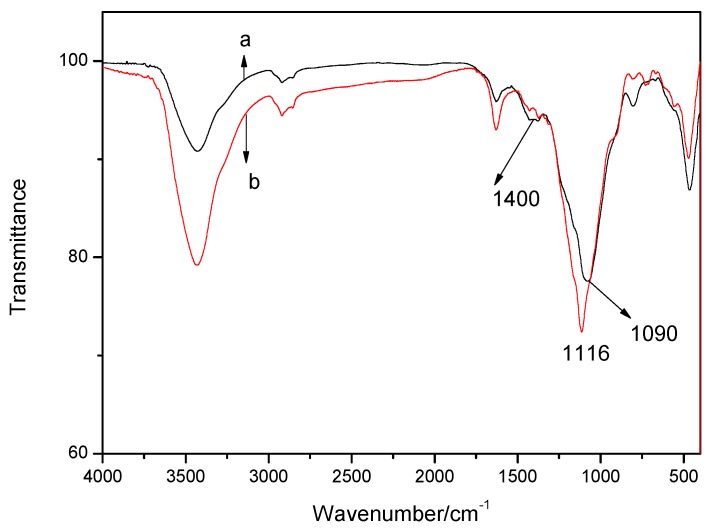
Infrared spectrum of carbon residue after cone calorimetry experiment: (a) PA66 composite with 15% ADCP; (b) PA66 composite with 3% nano-silica and 12% ADCP.

To illustrate how the formed coke affected the combustion of PA66/ADCP/nano-silica composites, the structure of the coke residue is further tested. Figure 6 showed the infrared spectrum of the burned coke residue after the cone calorimetry experiment. In the figure, 1116 and 1090 cm^−1^ were characteristic absorption peaks of the P = O bond, and 1400 cm^−1^ was the stretching vibration peak of the Si–O bond. It proved the existence of nano-silica in the carbon layer. The infrared spectra proved the synergistic flame retardancy between nano-silica and ADCP. They also showed that 15% ADCP PA66 composites and 3% nano-silica and 12% ADCP PA66 composites produced phosphoric acid groups that were dehydrated and carbonized during thermal degradation, thereby promoting carbonization.

### 3.5. Analysis of Cooperative Flame-Retardant Mechanism

**Figure 7:**
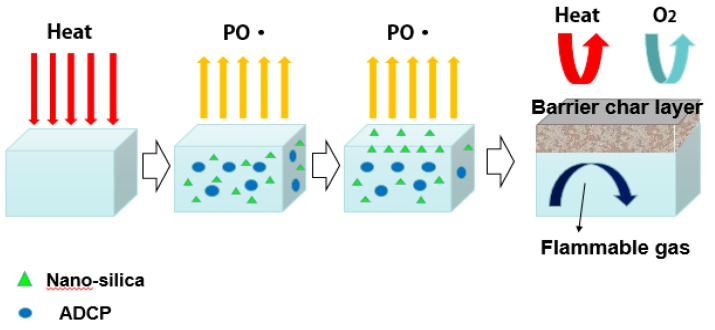
Analysis of flame-retardant mechanism

Figure 7 showed cooperative flame-retardant mechanism of ADCP and nano-silica. When an external heat source was applied to the PA66 composite material, the temperature of the composite material gradually rose and three physical states occurred: glass state, high elastic state, and viscous flow state. At the beginning of heating, the molecular chain of the composite material changed from frozen state to rotation state in the main chain single bond. The segmental conformation changed continuously, and the segment slipped. However, the main chain still could not move at this time. As the temperature increased further, the molecules could also slide relative to one another, marking the beginning of the viscous flow state [5]. As heat was applied to the polymer, the polymer absorbed energy and conducted it internally. The heat applied continuously accumulated on the surface of the material lowing to the low thermal conductivity of the polymer, and the composite decomposed and vaporized. The product that began to decompose was water, a small molecule of a compounding agent or a low molecular-weight polymer during processing. As temperature increased, the accumulated heat caused the weak bond of the composite chain to break. The first to break was the side group on the main chain. The cleavage gradually caused the chemical bonds in the main chain to break, and the structure of the main chain was retained in the form of carbon to form a carbon layer [20,21]. As the temperature increased, dicyclohexenyl aluminum hypophosphite was thermally decomposed to form phosphorus oxyacid, which catalyzed the dehydration of hydroxyl group-containing PA66 to form a carbon layer. It produced PO ·, which captured high-energy free radicals HO · and H · in the flame region, thereby suppressing free-radical reactions, asshownin Formula (1).

PO · + H · → HPO HPO · + H · → H_2_ + PO · PO · + OH · → HPO + · O ·(1)

The added nano-silica promoted flame retardancy in the condensed phase, which increased the density and the effectiveness of the carbon layer in preventing heat, flammable gases, and oxygen from entering the flame zone. It could attach to large molecules or was surrounded by large molecules due to its tiny structure, which made it to move with large molecules easily at high temperatures. When nano-silica migrated to the material surface in this way, it participated in the formation of a carbon layer, thereby improving the antioxidant properties of the carbon layer and protecting the internal matrix. It could synergizethe flame retardant. From a physical point of view, the dense carbon layer formed via synergistic flame retardation isolated the internal undecomposed PA66 from the high temperature and aerobic environment, thereby slowing the transfer of heat to the material and isolating the contact of oxygen with the flammable gas [21]. From a chemical point of view, the flammable gas interacted with the hot carbon layer to further oxidize or decompose, and the carbon layer absorbed heat through the decomposition reaction, thereby reducing heat transfer [1]. Nano-silica could react with carbon to produce good chemical heat shielding effect via decomposition endotherm under high-temperature conditions, such as in Formula (2) (kJ/mol, ΔH0).

SiO_2_(s) + C(s) → SiO(s) + CO(g) + 628.5 SiO_2_(s) + 2C(s) → Si(I) + 2CO(g) + 644.3 SiO_2_(s) + 3C(s) → SiC(s) + 2CO(g) + 512.6 SiC(s) + 2SiO_2_(s) → 3SiO(g) + CO(g) + 1372.9 SiO_2_(s) + Si(I) → 2SiO(s) + 614.7 (2)

In summary, the flame retardant ADCP can effectively promote the formation of a porous carbon layer on the surface of PA66 combustion, while nano-silica can increase the density of the carbon layer structure. Carbon layer mainly insulates heat and air, thereby providing a flame-retardant effect. The flame-retardant mechanism of ADCP mainly manifests in two aspects, condensed phase flame retardant and gas phase flame retardant. The flame-retardant mechanism of adding nano-silica mainly promotes the formation of the condensed phase. In contrast to the flame-retardant system with nano-silica added, the loose rough carbon layer formed by PA66 without nano-silica causes the internal flammable gas to enter the flame region through the pore structure. Hence, the effect is inferior to the addition of an appropriate amount of nano-silica. The compact and dense carbon layer structure can effectively prevent the entry of heat, flammable and combustion-supporting gas into the flame zone as well as the generation of molten droplets and flame propagation. Therefore, nano-silica can form an efficient flame-retardant system with ADCP.

## 4. Conclusions

The synergistic flame-retardant effect between dicyclohexenyl aluminum hypophosphite and nano-silica is studied in this work.The optimum addition ratio of dicyclohexenyl aluminum hypophosphite and nano-silica is 12:3 (weight ratio), which is determined via LOI and horizontal vertical burning test (UL-94). The synergistic flame-retardant effect of dicyclohexenyl aluminum hypophosphite and nano-silica is better than the single flame-retardant system. The synergistic flame-retardant mechanism is the high carbon content contributed by dicyclohexenyl aluminum hypophosphite to the formation of a carbon layer while nano-silica increases the compactness of the carbon layer structure.

## Figures and Tables

**Table 1:** Formulation and flame retardancy of PA66 and its composites.

Sample	PA66	ADCP	Nano-Silica	LOI	UL-94	
	(wt%)	(wt%)	(wt%)	(%)	Dripping	Rating
PA66-0	100	0	0	21.5	Y	V-2
PA66-1	85	15	0	32	N	V-0
PA66-2	88	12	0	30.5	N	V-0
PA66-3	87	12	1	31.7	N	V-0
PA66-4	85	12	3	33.1	N	V-0
PA66-5	83	12	5	35.2	N	V-0

**Table 2:** Peak mass loss rate and peak smoke rate of PA66 and its composites.

	PA66	15%ADCP FR-PA66	12%ADCP/3% Nano-Silica FR-PA66
Peak MLR(g/s)	0.13	0.08	0.06
Peak SPR(m^2^/s)	0.16	0.12	0.09
